# The effects of lighting conditions and food restriction paradigms on locomotor activity of common spiny mice, *Acomys cahirinus*

**DOI:** 10.1186/1740-3391-10-6

**Published:** 2012-09-09

**Authors:** Christopher C Chabot, Devin M Connolly, Brenda B Waring

**Affiliations:** 1 Department of Biological Sciences, Plymouth State University, Plymouth, NH 03264, UK

**Keywords:** (3-10) Circadian, Food entrainable oscillator, Food anticipatory activity, Constant light, Aschoff’s rule, Locomotor

## Abstract

**Background:**

An endogenous circadian clock controls locomotor activity in common spiny mice (Acomys cahirinus). However, little is known about the effects of constant light (LL) on this activity or about the existence of an additional food entrainable clock. A series of experiments were performed to investigate the effects of LL and DD on tau and activity levels.

**Methods:**

Spiny mice were housed individually and their running wheel activity monitored. One group of mice was exposed to LD, DD and several intensities of LL. Another group was exposed to a restricted feeding (RF) paradigm in light: dark (LD) during one hour before the L to D transition. Significance of rhythmicity was assessed using Lomb-Scargle periodograms.

**Results:**

In LD all animals exhibited nocturnal activity rhythms that persisted in DD. When animals were exposed to RF (during L), all of these animals (n = 11) demonstrated significant food anticipatory activity as well as an increase in diurnal activity. This increase in diurnal activity persisted in 4/11 animals during subsequent ad libitum conditions. Under LL conditions, the locomotor rhythms of 2/11 animals appeared to entrain to RF. When animals were exposed to sequentially increasing LL intensities, rhythmicity persisted and, while activity decreased significantly, the free-running period was relatively unaffected. In addition, the period in LL was significantly longer than the period in DD. Exposure to LL also induced long-term changes (after-effects) on period and activity when animals were again exposed to DD.

**Conclusions:**

Overall these studies demonstrate clear and robust circadian rhythms of wheel-running in A. cahirinus. In addition, LL clearly inhibited activity in this species and induced after-effects. The results also confirm the presence of a food entrainable oscillator in this species.

## Background

Virtually all organisms exhibit circadian rhythms that can be entrained by a light-dark (LD) cycle [1]. These LD cycles entrain the primary circadian pacemaker, which is often called the light-entrainable oscillator (LEO). This oscillator has been well studied in many mammalian species including rats [2], flying squirrels [3], weasels and mink [4]. Although LEOs have been the most thoroughly studied, over the past three decades evidence has been accumulating that suggests the presence of a multioscillatory system in many species. One oscillator in particular, the food-entrainable oscillator (FEO), has been studied in several species. For example, honeybees [5] display food anticipatory activity (FAA) associated with restricted food availability. When the bees were subjected to one bout of sugar water each day, they quickly learned to visit the food source either during or just before food availability. Similar instances of time-place association have been demonstrated in starlings [6], *Sturnus vulgaris*, and garden warblers [7], *Sylvia borin*. Furthermore, rats exposed to restricted daily feeding schedules exhibit increased activity and lever-pressing just before the scheduled feeding time(s) in both LD and LL. This activity can even persist for up to five days when food availability is completely eliminated [8]. Similar results demonstrating the presence of an FEO have been demonstrated in mice [9] and Syrian Hamsters [10]. Despite clear evidence from other species, the presence of FAA or a FEO has yet to be demonstrated in *A. cahirinus*.

Common spiny mice are known to be nocturnal in the lab [11] and in the field [12,13] and to exhibit clear wheel-running activity patterns that persist in constant conditions [14]. Results from numerous studies on many other species suggest that light has quantifiable effects on circadian rhythms such that for nocturnal animals, higher intensities of light result in decreased activity and increased period (*tau*) of their rhythm. This relationship is known in the field of circadian rhythms as “Aschoff’s rule” [1]. One species in which this relationship has not been investigated is the common spiny mouse, *Acomys cahirinus*, and this investigation was one of the goals in this study.

This series of experiments focused on the investigation of these issues in *A. cahirinus* and we hypothesized that (1) LL would cause activity changes consistent with Aschoff’s “rule” and (2) FAA would be present in both LD and LL, supporting the idea of a separate food-entrainable oscillator in this species.

## Methods

### Animals and environmental conditions

Mice were housed in standard laboratory plastic cages (20x20x40cm), each equipped with a running-wheel available to the mice at all times. Wheel turns were monitored with a physical switch and the activity stored on a computer (Effects of LL: DAM System, Trikinetics, Waltham, MA or Effects of RF: ClockLab, Actimetrics, Evanston, IL) for later analysis. Groups of six cages were held in light-tight wooden chambers (60x51x186cm) and lighting was provided by two four-foot fluorescent bulbs of 34 Watts producing 1400 Lux at cage height (Luna-Pro light meter; Gossen, Germany) in each chamber. The chambers were continuously ventilated using fans with temperature maintained at 24°C (±3°C) and humidity of 54% (± 5%). Each cage was always equipped with a water bottle that was refilled on a regular basis. When they were allowed to feed, the mice were given IAMS Chunks dog food.

### Experimental procedures

#### Effects of LD, DD and LL on activity rhythms

Mice (7-13 months) were originally purchased from a commercial supplier (Plymouth Pet & Aquarium; Plymouth, NH) and then bred in the laboratory at Plymouth State University’s Biology Department (Plymouth, NH). Same-sex mice were housed in pairs under 12:12 LD for at least 15 days prior to any experimentation. Then, at the start of each experiment, mice were individually housed and entrained to 12:12 LD for at least 10 days in cages with running wheels. In the first experiment, designed to determine *tau* in DD male mice (n = 10) were exposed to 55 days of constant dark (DD) after initial LD exposure of at least 10 days. A second experiment was designed to examine the effects of LL intensity on circadian wheel-running rhythms and to determine if exposure to constant light (LL) can cause “after effects” in *A. cahirinus*. These mice (n = 12; 6M, 6F) were exposed to two periods of DD (11 and 12 days respectively) sandwiched around one period of LL (23 days; 175 lux). Since no effects of sex were measured (p > 0.05, the data were combined for analyses. In a third experiment, male mice (n = 12) were exposed to four sequentially increasing LL intensities (66, 130, 350 and 1400 lux). Each intensity period lasted 21, 21, 32, and 25 days respectively. These experiments were carried out under the supervision of the Plymouth State University Institutional Animal Use and Care Committee.

#### Effects of food restriction paradigms

These experiments were designed to determine the effects of food restriction paradigms under LD and LL conditions on wheel-running activity of *A. cahirinus*. Eleven mice (3 female and 8 male; 4-9 weeks; again - no significant sex effects) were purchased from the same supplier or from the Laconia Pet Center (Laconia, NH). After exposure to at least 14 days of 12:12 LD (lights on 0800; lights off - 2000), a two hour period of daytime food restriction (RF) was implemented during L in the following way. At 1630, a metal feeder containing 40g of food was hung on the inside of each cage for a total of two hours and removed at 1830. Any excess food that had fallen into the cage was removed as well. The food remaining in the feeder and the cage was weighed and this value subtracted from the original amount to determine the total food consumed for that day per mouse. The next day at 1630, the feeders were again refilled with 40g of food and the process repeated for a total of 33 days. Animals never consumed all 40 g and indeed on average consumed much less (3.57+/-0.20g/day). In order to see if any RF associated activity would persist, the feeding schedule was then returned to the original 12:12 LD *ad libitum* conditions for 17 days. Then, the same RF method as described previously was implemented under constant light (LL) conditions for 28 days. Animal consumed significantly more food in LL (4.40+/-0.21g/day; P < 0.02). This was followed by *ad libitum* conditions imposed for 32 days in order to determine whether their rhythms had become entrained and a secondary food-entrainable clock was present.

### Data analysis

#### Effects of LD, DD and LL on activity rhythms

The running wheel data were collected by a computer data acquisition system (Drosophila Activity Monitor IV, Trikinetics Waltham, MA) and stored on a Macintosh Computer in five or ten minute intervals. RATMAN [15] was used to generate actograms from these files. Since activity data in experiment three were collected in ten minute intervals and RATMAN can only accept data in five minute intervals, ten minute interval data were equally divided into two five minute intervals. Alpha was calculated by using objectively (blind observer) drawn eye-fit lines on the actograms produced by RATMAN. RATWAVE was used to calculate *tau*[15] and the resulting *tau* was compared to the activity records. Ninety-five percent (96/105) values calculated by RATWAVE agreed very well with visual calculation. Five percent (9/105) produced values of *tau* that did not agree well with visual inspection. In these cases, *tau* was calculated using the slope of the eye-fit lines. This procedure had no effect on any statistical analysis.

Activity levels for each mouse was calculated by summing the number of wheel rotations per five minute intervals and then dividing by the total number of five minute intervals within each stage of the experiments. *Tau*, alpha, and total activity were calculated for all mice during each of the experiments. Analysis of variance (Super ANOVA, Abacus Concepts, Inc.) was used to determine overall effect (p < 0.05). Significant differences between means were determined by least square means method (p < 0.05).

#### Effects of food restriction paradigms

All running wheel data were collected and stored in 5-minute bins. Sequential actograms for each individual subject were visually inspected and Lomb-Scargle periodogram analysis was used to calculate tau (p < 0.05) and to determine the presence of significant rhythmicity [16]. Microsoft Office 2003 Excel (Redmond, WA) was used to calculate descriptive statistics and perform Student’s t-tests to determine overall significance (p < 0.05) between daytime and nighttime activity, as well as significant differences between normal and restricted feeding activity levels in LD and LL conditions. Activity in the 1h prior to food restriction was compared in LD and LL to determine if food anticipatory activity had occurred.

## Results

### Food restriction effects

**Figure 1:**
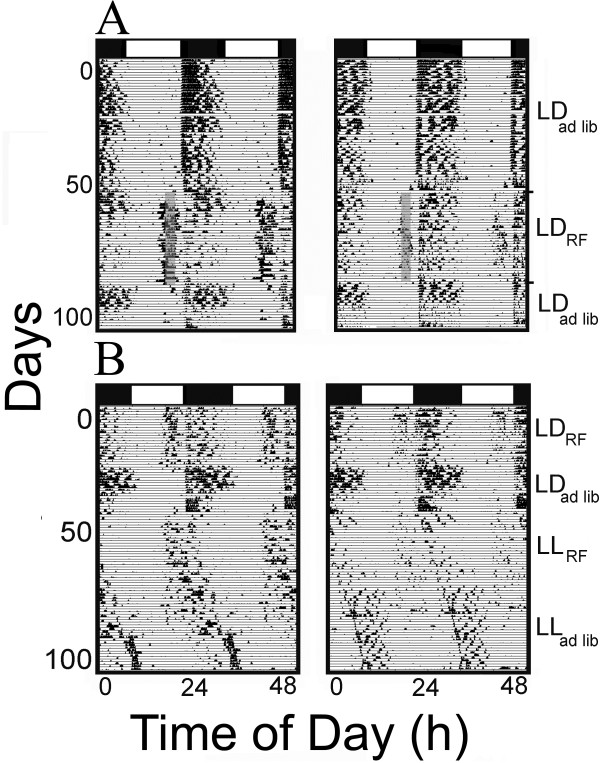
**FAA and FEO in *A. cahirinus*.** Representative actograms showing the effects of food restriction (RF) paradigms in LD (**A**) and LL (**B**) on running-wheel activity of four mice. The shaded grey region indicates a 2h period of restricted feeding (16:30 – 18:30 h).

The effects of food restriction paradigms (RF) in LD and LL conditions on running-wheel activity of four mice are represented in Figure 1. When the mice were exposed to 12:12 LD *ad libitum* conditions, all mice (11/11) individuals showed significant nocturnal activity (Figure 1A). When the LD entrained mice were then exposed to a two-hour period of RF during the daytime, 10/11 (91%) individuals still exhibited significantly more activity during the nighttime – i.e. – they were still generally nocturnally active. However, all (11/11) mice also showed a significant increase in daytime activity during the 1 hour prior to food availability. This increase in diurnal activity persisted in 4/11 animals when food restriction was discontinued and *ad libitum* conditions reinstated (Figure 1 - left panels).

When exposed to LL, the main activity bouts of most (9/11) animals exhibited no coordination with the restricted feeding (Figures 1B - right panel) while 2/11 animals appeared to entrain to the RF (Figure 1B - left panel). Neither activity levels nor *taus* were significantly different in either LL food restriction or LL *ad libitum* (t = 0.12; p > 0.05).

### LD and DD

**Figure 2:**
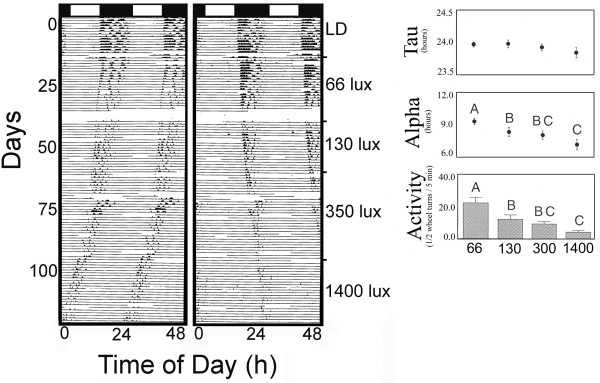
**Increasing LL causes decreasing activity.** Representative actograms (**A**) of two mice entrained to LD and then exposed to increasing LL intensities (66 to 1400 lux). **B**) – Overall results (N = 12).

The effects of LD and DD conditions on alpha, *tau* and running-wheel activity are shown in Figure 2. All animals (10/10) entrained to the LD and these rhythms persisted in DD with periods less than 24 hours. Alpha showed a significant lengthening over time (F (2,18) = 7.19; p < 0.02) in DD conditions (Figure 2B). Neither *tau* (F (2,18) = 1.53; p < 0.25) nor activity levels (F (2,18) = 0.38; p < 0.69) changed significantly over the 50+ days in DD.

### Aftereffects

**Figure 3:**
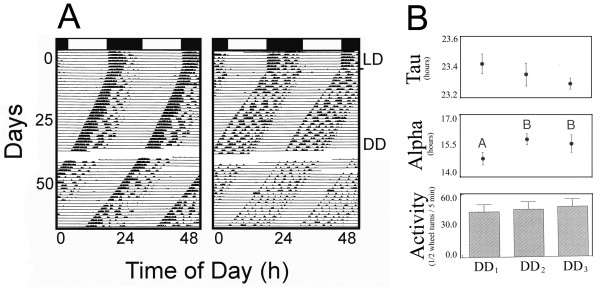
**LD entrained rhythms persist in DD.** Representative double-plotted actograms (**A**) and overall results (**B**) of two *A. cahirinus* exposed to 12:12 Light-Dark (LD) and then constant dark (DD). **A.** Black bars at the top signify the hours of dark; white bars signify hours of light. Each black tick mark indicates a bout of activity recorded of the individual animal. **B.** Graphical values represent the means ± the standard error of the means (N = 10). Means with different letters above hem are significantly different (LSM, P < 0.05). DD_1_ = 1^st^ 15 days in DD; DD_2_ = middle 15 days in DD; DD_3_ = last 15 days in DD.

The effects of an intervening LL (175 lux) on DD are presented in Figure 3. When animals (N = 12) were exposed to LL after DD_1_, a significant decrease in alpha was observed (F (2,22) = 11.18; p < 0.003; Figure 3B). When LL was subsequently changed to DD_2_ alpha increased significantly, but not to its original DD_1_ value. *Tau* was less than 24 hours in DD_1_ but became significantly longer (F (2,22) = 21.46; p < 0.0001) upon LL exposure. When the photic conditions reverted back to DD (DD_2_), there was a significant shortening in *tau* compared to both LL and DD_1_*taus*. In addition, activity significantly diminished from LL to DD_2_ (F (2,22) = 8.43; p < 0.003).

### LL Intensity

**Figure 4:**
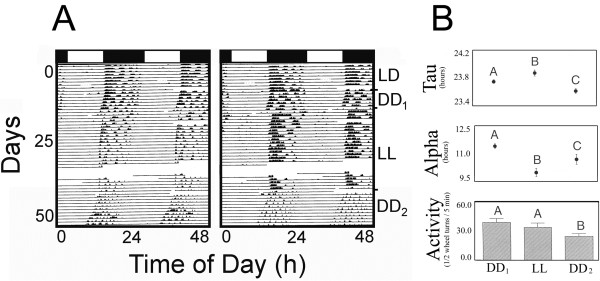
**LD induces “aftereffects”.** Representative actograms of two different mice (**A**) entrained to LD and then exposed to two periods of DD sandwiched around a period of constant light (LL; 175 lux). **B**) – overall results (N = 12).

The effects of increasing LL intensities (ranging from 66 to 1400 lux) on alpha, *tau* and activity levels are presented in Figure 4. As LL intensity increased, a significant decrease in both alpha (F (3,33) = 8.20; p < 0.0009) and running-wheel activity (F (3,33) = 18.86; p < 0.0001) occurred. However, *tau* was not significantly affected (Figure 4B).

## Discussion

### FAA in LD and LL

Our study is the first to provide evidence of food anticipatory activity (FAA) in the common spiny mouse. In LD, all animals significantly increased their wheel-running activity during the one hour prior to food availability and thus exhibited FAA in LD (Figure 1A). Similar responses to restricted food access have been seen in a wide variety of animals. Syrian hamsters [10], rats [8,17], rabbits [18], mice [19], predatory marsupials [20,21], and birds [6,7] all have been shown to exhibit FAA (but not squirrel monkeys, [22]).

### Entrainment in LD and LL - FEO

Our study is also the first to provide evidence of food entrainable oscillator (FEO) in the common spiny mouse. When RF was discontinued and food became freely available in LD, diurnal activity persisted for several cycles in 4/11 animals (Figure 1A - left panel), indicating the presence of a separate FEO in this species. Persistence of this activity demonstrates entrainment of an underlying clock and has been observed in rats [23] and some strains of mice but not others [24].

RF also effectively entrained the main activity bout in a small percentage of animals. In LL, two animals clearly synchronized to the restricted feeding as their activity increased during the one hour that would have been prior to food availability (Figure 1B - left panel). The remaining nine animals exhibited free-running activity and showed no coordination with the period of restricted feeding (Figure 1B - right panel). Rats [25] and Syrian hamsters – [26] have also demonstrated synchronization to feeding times in LL. RF is also an effective entraining signal for fetal spiny mice [14]. However, in stark contrast, food restriction paradigms failed to entrain activity rhythms in squirrel monkeys [27] and hamsters [28]. Likewise in some small carnivores, there is little evidence of an endogenous timing mechanism to synchronize feeding behavior [4].

Overall, in common spiny mice, restricted feeding was less effective in eliciting FAA in LL than in LD. These findings are similar to those in rats [8]. In addition, nearly all shoals of shiners showed FAA in LD whereas only 22% exhibited this behavior in constant conditions (DD) [29]. These authors suggested that while shiners may have an FEO, their RF activity may also be tied to an LEO. This hypothesis has been suggested for a number of animals [30] and could be the case for the common spiny mouse.

### Increasing diurnal activity

Daytime food availability also significantly increased daytime activity in this nocturnal animal; Figure 2). Interestingly, working for food apparently causes a switch from nocturnal to diurnal activity in mice [31]. In other nocturnal species, diurnal bouts of daytime activity has been shown to entrain a variety of tissues to the daily RF schedule [32-35]. This type of biphasic activity pattern has also been reported in field mice [19]. Interestingly another member of this genus, *A. russatus,* can completely shift its activity from nocturnal to diurnal activity in response to coexistence with *A. cahirinus*[36,37]. Shkolnik [37] reported that the two species compete for the same food and this temporal partitioning is a means to allow allopatry. Haim & Fluxman [38] have suggested that both the switch from nocturnal to diurnal activity and reduced activity of *A. russatus* is due to chemical signals released by *A. cahirinus*. Interestingly, they further report that this chemical induced shift in activity only occurs in a photoperiodic environment, not in constant conditions.

#### Nocturnal activity

In this study, *Acomys cahirinus* displayed robust activity rhythms in 12:12 LD conditions with a distinct nocturnal preference. These results are consistent with those previously reported in both the lab [11,39] and field [12]. Many other mammals such as the rat [40], Northern brown bandicoot [41], and mouse [42] also typify rhythms of activity that can be synchronized to LD cycles. When exposed to constant darkness (DD), the nocturnal activity rhythms of *A. cahirinus* persisted with periods of less than 24 hours. This persistence of activity rhythms in constant conditions is indicative of endogenous circadian control of locomotor activity by a light-entrainable oscillator (LEO). Weaver and Reppert [14] also found that activity rhythms of *A. cahirinus* persisted in DD, lending further support to the presence of a LEO oscillator in this species.

##### LL

When animals were exposed to LL, there were significant effects of light intensity on free running period, alpha and overall activity. Upon exposure to increasing LL intensities, *A. cahirinus* exhibited a significant decrease in locomotor activity. Thus, these findings support one aspect of “Aschoff’s rule” that, in nocturnal animals, higher intensities of light result in decreased activity and alpha [1]. Similar findings in the field were recently reported by Rotics et al., [13] in this species. When *A. cahirinus* were kept in outdoor enclosures and exposed to nocturnal illumination, they decreased their nocturnal activity. While the authors suggested that this decrease in nocturnal activity could have been due to increased predation pressures, our results, in the absence of any predators, suggests a more direct light effect on the circadian system. In addition, when mice were exposed to LL after being exposed to DD, *tau* lengthened significantly (Figure 2; [43]). However, while we did not find significant effects of LL intensity on *tau*, there was a trend of decreasing *tau* with increasing LL intensity. Thus, an increase in the “n” may have produced statistically significant results. Cohen and Kronfeld-Schor [44] recently reported increases in *tau* in *A. russatus* when this species went from DD (23.72h) to LL (24.47h). We report here more modest increases (from 23.25 to 23.9h) in *A. cahirinus*. We also report apparent “aftereffects” of LL on *tau* (Figure 2) as reported in several nocturnal rodent species [43].

## Conclusions

Overall these studies demonstrate clear and robust circadian rhythms of wheel-running in *A. cahirinus*. In addition, LL clearly inhibited activity in this species and induced after-effects. The results also confirm the presence of a food entrainable oscillator in this species further extending our understanding of this important issue.

## Abbreviations

LL: Constant light; DD: Constant darkness; FEO: Food entrainable oscillator; FAA: Food anticipatory activity.

## Competing interests

The authors declare that they have no competing interests for this manuscript.

## Authors’ contributions

DMC helped to conceive of and carry out the food restriction studies and drafted the manuscript. BBW helped to conceive of and carry out the LL and DD studies. CCC helped to conceive of the study and participated in its design and coordination and helped to draft the manuscript. All authors read and approved the final manuscript.
